# A small-molecule TrkB ligand restores hippocampal synaptic plasticity and object location memory in Rett syndrome mice

**DOI:** 10.1242/dmm.029959

**Published:** 2017-07-01

**Authors:** Wei Li, Alba Bellot-Saez, Mary L. Phillips, Tao Yang, Frank M. Longo, Lucas Pozzo-Miller

**Affiliations:** 1 Department of Neurobiology, Civitan International Research Center, University of Alabama at Birmingham, Birmingham, AL 35294, USA; 2 Department of Neurology and Neurological Sciences, Stanford University School of Medicine, Stanford, CA 94305, USA

**Keywords:** BDNF, LM22A-4, MeCP2, LTP, Voltage-sensitive dye imaging, Ca^2+^ imaging, Object location memory, CA1 pyramidal neuron

## Abstract

Rett syndrome (RTT) is a neurodevelopmental disorder caused by mutations in methyl-CpG-binding protein-2 (*MECP2*), a transcriptional regulator of many genes, including brain-derived neurotrophic factor (*BDNF*). BDNF levels are reduced in RTT autopsy brains and in multiple brain areas of *Mecp2*-deficient mice. Furthermore, experimental interventions that increase BDNF levels improve RTT-like phenotypes in *Mecp2* mutant mice. Here, we characterized the actions of a small-molecule ligand of the BDNF receptor TrkB in hippocampal function in *Mecp2* mutant mice. Systemic treatment of female *Mecp2* heterozygous (HET) mice with LM22A-4 for 4 weeks improved hippocampal-dependent object location memory and restored hippocampal long-term potentiation (LTP). Mechanistically, LM22A-4 acts to dampen hyperactive hippocampal network activity, reduce the frequency and amplitude of miniature excitatory postsynaptic currents (mEPSCs), and reduce the frequency of spontaneous tetrodotoxin-resistant Ca^2+^ signals in *Mecp2* mutant hippocampal neurons, making them comparable to those features observed in wild-type neurons. Together, these observations indicate that LM22A-4 is a promising therapeutic candidate for the treatment of hippocampal dysfunction in RTT.

## Abstract (precis)

**Editors' choice:** The brain-penetrant BDNF loop domain mimetic LM22A-4 improves synaptic plasticity and spatial discrimination memory in Rett syndrome mice, making it a promising therapeutic candidate for the treatment of hippocampal dysfunction.

## INTRODUCTION

Rett syndrome (RTT) is an X chromosome-linked neurodevelopmental disorder that affects approximately 1:10,000 females worldwide (Neul et al., 2010). RTT individuals develop typically until 6-18 months, when neurological symptoms, including intellectual disability, autistic features, deficits in motor control and sensory perception, breathing irregularities and epilepsy disorders begin (Percy, 2011). The majority of RTT individuals carry loss-of-function mutations in methyl-CpG-binding protein-2 (*MECP2*), which encodes the transcriptional regulator MeCP2 (Amir et al., 1999). *Mecp2-*deficient mice recapitulate several neurological features of RTT, including impaired hippocampal-dependent learning and memory (Calfa et al., 2011b; Li and Pozzo-Miller, 2012), which makes them useful experimental models for preclinical studies (Katz et al., 2012).

One prominent target of MeCP2 transcriptional regulation is the gene encoding brain-derived neurotrophic factor (BDNF) (Chahrour et al., 2008; Chen et al., 2003; Martinowich et al., 2003), a neurotrophin that plays critical roles in neuronal survival, differentiation, and synapse formation and plasticity (Park and Poo, 2013). BDNF levels are lower in RTT autopsy brains (Abuhatzira et al., 2007; Deng et al., 2007) and *Mecp2*-deficient mice (Chang et al., 2006; Li et al., 2012; Schmid et al., 2012; Wang et al., 2006). Since *Bdnf* overexpression in male *Mecp2* knockout (KO) mice rescues several RTT-like neurological and motor symptoms (Chang et al., 2006), enhancement of BDNF signaling is considered a potentially useful therapeutic approach for RTT (Katz, 2014). Owing to its low blood-brain barrier permeability that limits the bioavailability of peripherally administered BDNF, therapeutic approaches have relied on BDNF ‘mimetics’. One approach involved the use of ampakines, which are known to increase *Bdnf* expression by their action on AMPA-type glutamate receptors (Lauterborn et al., 2000). Peripheral treatment with ampakines significantly improved respiratory dysfunction in male *Mecp2* KO mice (Ogier et al., 2007). A more direct approach is to activate TrkB receptors with small-molecule mimetics of the BDNF loop domain that are designed *in silico* to interact with their BDNF binding pocket (Massa et al., 2010). One such TrkB ligand with partial agonist activity, LM22A-4, reduced synaptic hyperactivity within respiratory centers in the brainstem, and improved respiratory function in female *Mecp2* heterozygous (HET) mice (Kron et al., 2014; Schmid et al., 2012).

Here, we describe how a 4-week systemic LM22A-4 treatment in symptomatic female *Mecp2* HET mice improved general phenotype, motor activity and hippocampal-dependent object location memory by activating the BDNF receptor TrkB. Long-term potentiation (LTP) of hippocampal excitatory synaptic transmission, the cellular substrate of learning and memory, was also restored by LM22A-4 treatment in *Mecp2* HET mice. Furthermore, LM22A-4 reduced the spatio-temporal spread of neuronal depolarizations in hippocampal slices from *Mecp2* HET mice to levels comparable to wild-type (WT) littermates, thus preventing network hyperactivity. In addition, LM22A-4 reduced the frequency and amplitude of miniature excitatory postsynaptic currents (mEPSCs) in CA1 pyramidal neurons of organotypic slices from *Mecp2* KO mice. Finally, LM22A-4 reduced the frequency of spontaneous Ca^2+^ signals, reflecting quantal transmitter release in cultured hippocampal *Mecp2* KO neurons. Together, these observations indicate that LM22A-4 is a promising therapeutic candidate for the treatment of hippocampal dysfunction in RTT.

## RESULTS

### LM22A-4 improves general phenotypes, motor activity and hippocampal-dependent memory in female *Mecp2* heterozygous mice

Because RTT occurs primarily in females, we chose to study the effects of a chronic peripheral treatment with LM22A-4 in female *Mecp2* HET mice. In additional experiments, we used male *Mecp2* KO mice that are homogenously deficient in MeCP2 expression to investigate the underlying cellular mechanisms of LM22A-4 actions. Following an established and successful dosing regime (Kron et al., 2014; Schmid et al., 2012), 4-month-old female *Mecp2* HET mice and age-matched WT littermates received intraperitoneal (i.p.) injections of LM22A-4 (50 mg/kg) twice daily for 1 or 2 months. During the treatment, weight and general phenotypes were assessed every week. At the end of the treatment, animals were examined for motor function and then sacrificed under deep anesthesia for assessment of activation of TrkB receptors and their downstream signaling.

**Fig. 1.**
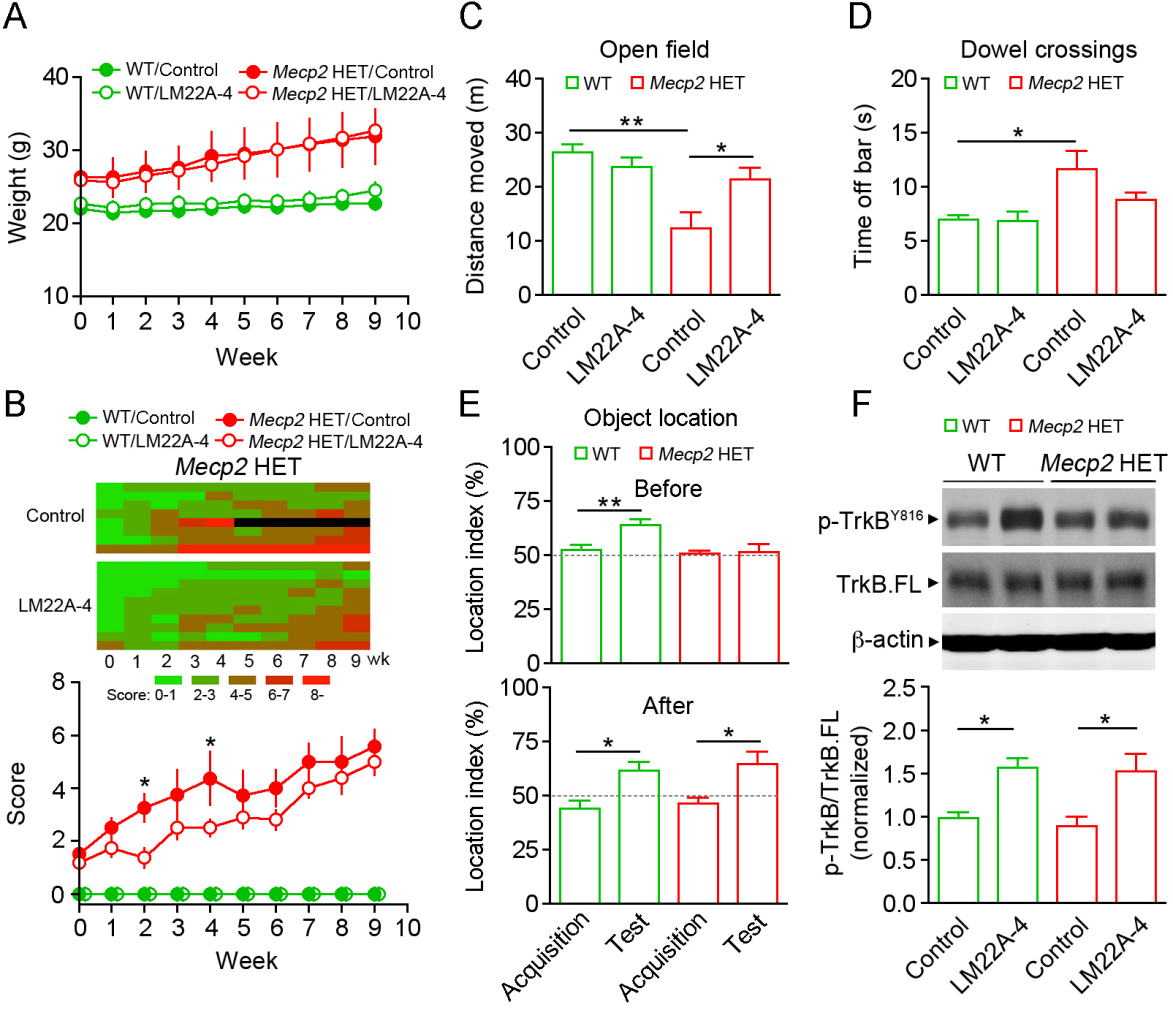
**LM22A-4 improves general phenotypes in *Mecp2* HET mice.** (A) Body weight measured every week in female WT and *Mecp2* HET mice during treatment with vehicle or LM22A-4. (B) Progression of average phenotype scores in WT and *Mecp2* HET mice treated with vehicle or LM22A-4. Inset shows an individual raster plot of score progression in *Mecp2* HET control (top) and LM22A-4 (bottom) groups. Black denotes death of that mouse. (C) Average distance traveled in a 60×60 cm arena during a 10 min period. (D) Average falling latency from an elevated dowel. (E) WT and *Mecp2* HET mice were exposed to two identical objects for 5 min during the acquisition phase, and 2 h later underwent the test trial in which one object was moved to a new location. Object location test was carried out before (top) and after (bottom) 1 month treatment with vehicle or LM22A-4. (F) Quantitative analyses of the levels of phosphorylated TrkB (p-TrkB^Y816^) relative to the total full-length (TrkB.FL) protein in hippocampal homogenates. Top panel shows representative examples of western immunoblots for p-TrkB^Y816^, TrkB.FL and the internal control β-actin. Data are mean±s.e.m. *P<0.05, **P<0.01.

Female *Mecp2* HET mice of the Jaenisch strain typically exhibit an increase in body weight during the symptomatic stage (Chen et al., 2001) (Fig. 1A; WT control, *n*=10; *Mecp2* HET control, *n*=9). LM22A-4 treatment had no significant effect on body weight in WT and *Mecp2* HET mice (Fig. 1A; WT LM22A-4, *n*=10; *Mecp2* HET LM22A-4, *n*=11). We also scored RTT-like general symptoms consisting of ill-groomed condition, reduced motility, imbalanced gait, hindlimb clasping, irregular breathing and tremor (Guy et al., 2007). Each category was scored (0: absent; 1: moderate; 2: severe), and the aggregated value showed overall phenotypes. LM22A-4 significantly reduced the score value in *Mecp2* HET mice especially during the early phase of the treatment (Fig. 1B; *P*<0.001, two-way repeated measures ANOVA), but did not affect WT mice.

To assess general locomotor activity, we performed an open field test and measured the distance traveled during a 10 min period. We found that *Mecp2* HET mice traveled significantly shorter distances than WT mice (Fig. 1C; WT control, *n*=9; *Mecp2* HET control, *n*=7; *P*<0.01, one-way ANOVA), and that LM22A-4 treatment increased it to levels comparable with the WT (*n*=7; *P*<0.05 vs *Mecp2* HET control). However, LM22A-4 had no effect on the distance traveled by WT mice (*n*=9). Next, we performed dowel crossings to evaluate motor coordination. *Mecp2* HET mice took longer than the WT control to walk off the elevated dowel (Fig. 1D; *P*<0.05, one-way ANOVA), which was slightly reduced by LM22A-4 treatment.

In a different set of experiments, we performed the object location task to evaluate hippocampal-dependent spatial memory (Barker and Warburton, 2011). During the acquisition phase, animals normally show equal preference for two objects that are placed in an arena, while they exhibit a higher preference for the moved object during the subsequent test phase. The object location memory can thus be estimated by calculating the location preference index (time spent on moved objects over total time spent on two objects). As expected, the preference index in female WT mice was larger for the moved object during the test phase than the acquisition phase (Fig. 1E, top; *n*=7; *P*<0.01, paired *t*-test). However, we did not observe any increase in *Mecp2* HET mice (*n*=7), suggesting an impairment of objection location memory. Interestingly, systemic LM22A-4 treatment for 4 weeks significantly enhanced the preference index for the moved object in *Mecp2* HET mice (Fig. 1E, bottom; *n*=7; *P*<0.05, paired *t*-test), but was unable to further promote it in WT mice (*n*=7).

To confirm the central target engagement of peripherally administered LM22A-4, we measured the activation state of TrkB receptors and their downstream signaling. Hippocampal homogenates from mice treated with LM22A-4 for 2 months were subject to western immunoblotting. We found a significant increase in the ratio of phosphorylated TrkB^Y816^ to full-length TrkB in both WT and *Mecp2* HET hippocampi after LM22A-4 treatment (Fig. 1F; *n*=8 each; *P*<0.05, one-way ANOVA). Consistently, the ERK and PLCγ cascades were upregulated in WT samples (Fig. S1A; *P*<0.001) and the PLCγ signaling was activated in *Mecp2* HET mice (*P*<0.05). We also measured the acute effects of LM22A-4 on TrkB phosphorylation in hippocampal slices prepared from male *Mecp2* KO mice, where every neuron lacks MeCP2. Interestingly, the levels of phosphorylated TrkB increased significantly after a 30 min exposure to LM22A-4 (500 nM), but only in *Mecp2* KO slices (Fig. S1B, left; *Mecp2* KO control, *n*=14; *Mecp2* KO LM22A-4, *n*=13; *P*<0.001) and not in WT slices (WT control and LM22A-4, *n*=16 each), which is consistent with the actions of a partial agonist (Massa et al., 2010). Unlike LM22A-4, the full agonist BDNF (250 ng/ml) activated TrkB receptors in slices from both WT (Fig. S1B, right; *n*=4; *P*<0.05) and *Mecp2* KO mice (*n*=4; *P*<0.01). The effect of LM22A-4 on TrkB phosphorylation was completely blocked by the pan-Trk inhibitor K252a (200 nM) (*n*=12; *P*<0.01 vs LM22A-4). Treatment with K252a alone had no significant effect on the ratio of phosphorylated TrkB to full-length TrkB (*Mecp2* KO K252a, *n*=6). Additionally, acute LM22A-4 application did not alter TrkB downstream signaling in either genotype (Fig. S1C). Collectively, these data suggest that LM22A-4 improves general phenotype, motor activity and object location memory in *Mecp2* HET mice by activating TrkB signaling in the brain.

### LM22A-4 restores hippocampal LTP in female *Mecp2* heterozygous mice

**Fig. 2.**
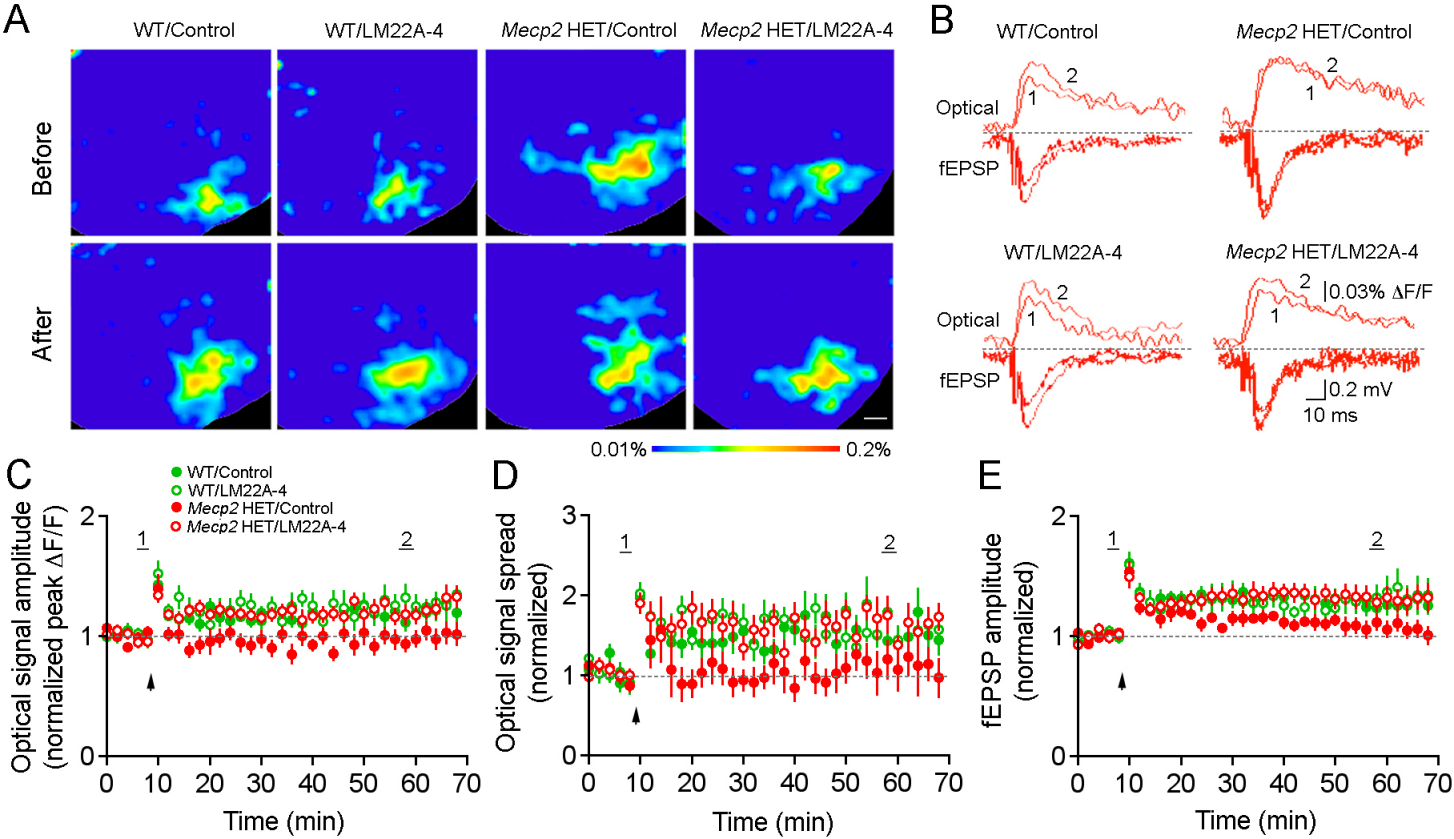
**LM22A-4 improves LTP at CA3-CA1 synapses in hippocampal slices from female *Mecp2* HET mice.** (A) Representative images of VSD signals before (top) and 50 min after (bottom) induction of TBS-LTP in WT and *Mecp2* HET mice treated with vehicle or LM22A-4. A single stimulation pulse was delivered to Schaffer collaterals to evoke fEPSPs and VSD signals in area CA1. (B) Representative traces showing simultaneously recorded VSD signals and field EPSPs before and after TBS-LTP induction. Numbers indicate the time in average plots shown in panels C-E. (C-E) The time course of average VSD signal peak amplitude (C) and spatial spread (D), and field EPSP amplitude (E) during LTP induction in WT and *Mecp2* HET mice treated with vehicle or LM22A-4. Arrowheads indicate the start of TBS. Scale bar: 300 µm. Data are mean±s.e.m.

We next evaluated the effect of a 2 month treatment with LM22A-4 on LTP at excitatory synapses in the hippocampus, the cellular substrate of learning and memory. We recorded subthreshold excitatory postsynaptic potentials (fEPSPs) at CA3-CA1 synapses and simultaneously imaged voltage-sensitive dye (VSD) signals throughout the entire hippocampal slice (Fig. 2A,B) (Chang and Jackson, 2006; Li et al., 2016). In slices from control WT mice, the peak amplitude and spatial spread of VSD signals in area CA1 that were evoked by a single afferent stimulation to the Schaffer collaterals increased significantly after induction of LTP with a theta burst stimulus (TBS), following a time course similar to that of the simultaneously recorded field EPSPs (Fig. 2C-E; *n*=12 slices/10 mice; *P*<0.01 baseline vs 50 min after TBS, Student's *t*-test). This LTP of VSD signal amplitude and spatial spread, and of fEPSPs in WT mice was not affected by LM22A-4 treatment (Fig. 2C-E; *n*=12/9). Similar to male *Mecp2* KO mice (Li et al., 2016), TBS stimulation failed to induce LTP of VSD signals and fEPSPs at CA3-CA1 synapses of female HET mice (Fig. 2C-E; *n*=10/9). Consistent with the improvement of hippocampal-dependent memory, LM22A-4 restored LTP of both VSD signals and fEPSPs in *Mecp2* HET mice (*n*=15/10; *P*<0.01), which were comparable to those in WT mice treated with LM22A-4. Together, these data demonstrate that systemic LM22A-4 treatment restores LTP at hippocampal synapses in female *Mecp2* HET mice.

### LM22A-4 reduces synaptic strength and network hyperactivity in the hippocampus of female *Mecp2* heterozygous mice

**Fig. 3.**
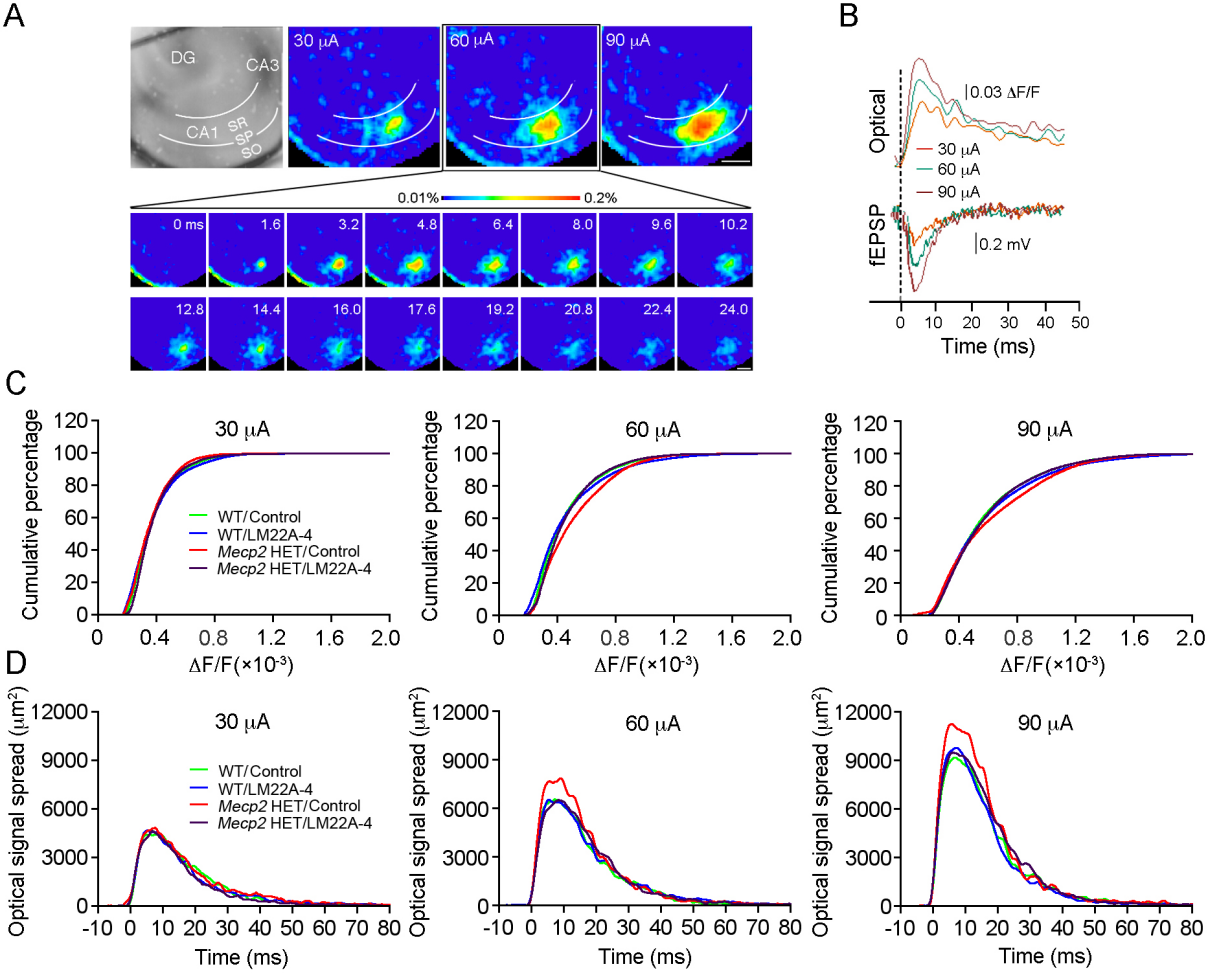
**LM22A-4 reduces network hyperactivity in *Mecp2* HET mice.** (A) Representative images of VSD signals evoked by stimulation of Schaffer collaterals with single pulses of 30, 60 and 90 µA in a WT control slice. Inset shows the time-lapse presentation of VSD signals in response to a 60 µA stimulus. SR, stratum radiatum; SP, stratum pyramidale; SO, stratum oriens; DG, dentate gyrus. (B) Representative traces of simultaneously recorded VSD signals and field EPSPs evoked by 30, 60 and 90 µA stimuli. (C) Cumulative percentage of VSD signal amplitude evoked by 30, 60 and 90 µA stimuli in slices from WT and *Mecp2* HET mice treated with vehicle or LM22A-4. VSD signals are defined as those that are beyond 2-times the standard derivation of background noise. (D) Spatiotemporal spread of VSD signals evoked by afferent stimulation at 30, 60 and 90 µA intensities. Scale bars: 300 µm. All data in B-D are means.

The lack of LTP at hippocampal synapses of male *Mecp2* KO mice results from the saturation of their plasticity range by already potentiated synapses (Li et al., 2016). To determine if this is also the case in *Mecp2* HET mice, and if LM22A-4 restores LTP by reducing synaptic strength allowing for synaptic potentiation, we performed VSD imaging and obtained the input-output (I-O) relationship of fEPSPs and VSD signals (Fig. 3A). VSD signals in CA1 evoked by increasingly stronger stimulation of Schaffer collaterals are proportional and have similar kinetics to fEPSP (Fig. 3B). VSD signals larger than two-times the standard derivation of background noise were used to calculate the cumulative percentage of VSD signal amplitudes. Compared with WT control slices (*n*=18/10), the cumulative percentage of VSD signal amplitudes was significantly shifted to larger amplitudes in *Mecp2* HET slices, indicating that excitatory synapses are stronger in female *Mecp2* HET mice (Fig. 3C; *n*=12/9; *P*<0.001, K-S test), as reported for male *Mecp2* KO mice (Calfa et al., 2011a; Li et al., 2012, 2016). LM22A-4 treatment did not affect VSD signals in WT mice (*n*=20/9), but it significantly shifted the cumulative percentage of VSD signal amplitudes to lower levels in *Mecp2* HET slices, making it comparable to WT slices (*n*=16/10; *P*<0.001 vs *Mecp2* HET control; *P*>0.05 vs WT LM22A-4). Similar results were obtained for the cumulative percentage of the spatial spread of VSD signals (Fig. 3D).

**Fig. 4.**
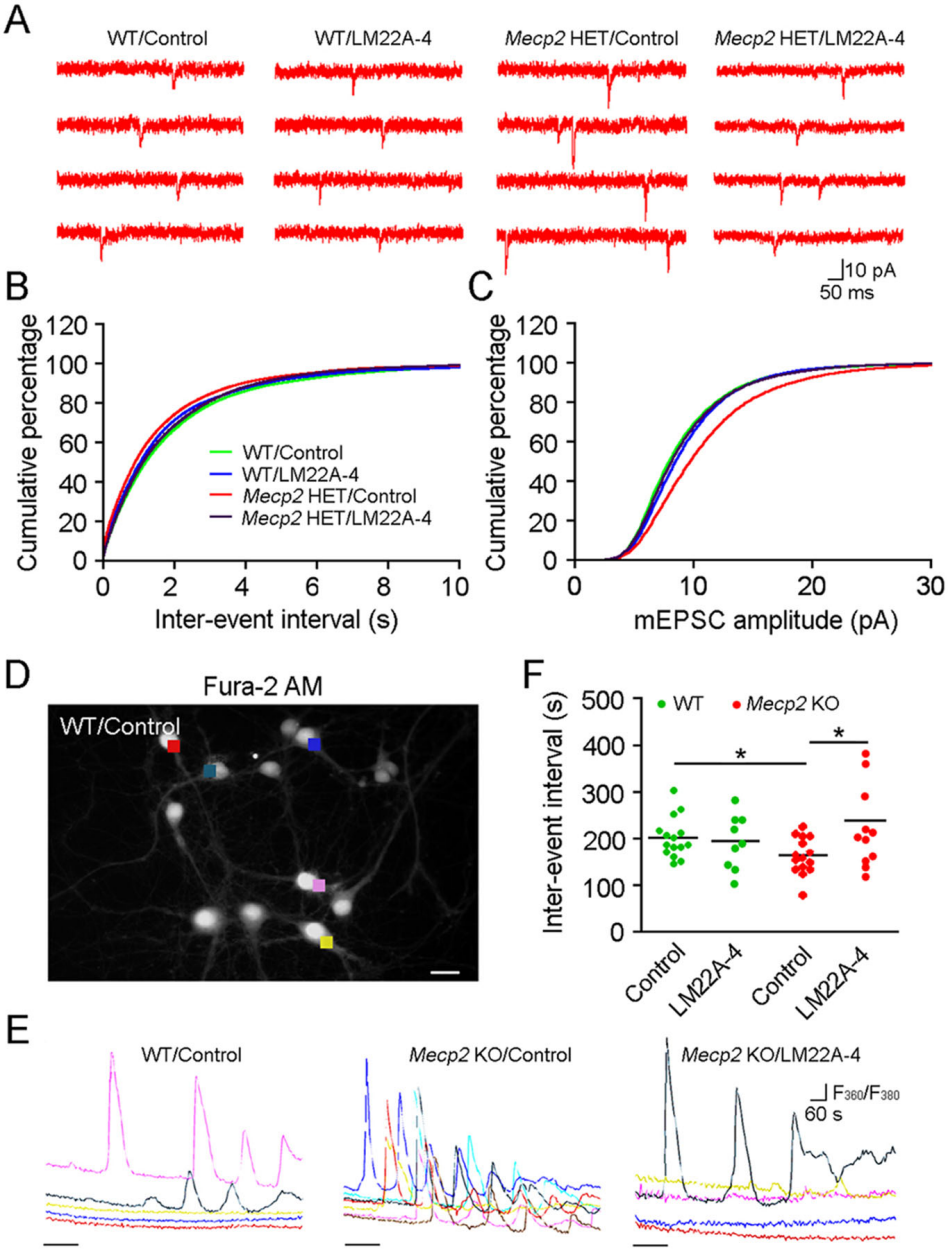
**LM22A-4 modulates excitatory quantal synaptic transmission and spontaneous Ca^2+^ transients (SCTs) in hippocampal neurons from male *Mecp2* KO mice.** (A) Representative traces of mEPSCs recorded from CA1 pyramidal neurons in hippocampal slice cultures in the presence of TTX (1 µM) and picrotoxin (50 µM). Cultured WT or *Mecp2* KO slices (DIV 10-12) were treated with vehicle or LM22A-4 (500 nM) for 2 days. (B,C) Cumulative percentage of the IEI (B) and amplitude (C) of mEPSCs in WT or *Mecp2* KO slices. (D) Representative view of WT neurons stained with Fura-2 AM (5 µM). Background-subtracted fluorescence intensity measurements were obtained with regions of ROIs defined over the somatodendritic area of individual neurons (colored squares). (E) Representative spontaneous SCTs obtained from WT and *Mecp2* KO neurons in response to puffing of aCSF or LM22A-4 (500 nM) for 2 min. Color-coded traces in the WT/control group were derived from neurons in D. (F) Average IEI of SCTs in WT and *Mecp2* KO neurons treated with aCSF or LM22A-4. Scale bar: 100 µm. All data in B,C,E are means. *P<0.05.

To directly test the effects of LM22A-4 on synaptic strength, we recorded mEPSCs from CA1 pyramidal neurons in organotypic slice cultures treated with LM22A-4 for 2 days. Whole-cell intracellular recordings were performed in the presence of the sodium channel blocker TTX and the GABA_A_R antagonist picrotoxin (Fig. 4A). Compared with WT neurons (*n*=9 cultured slices/3 mice), *Mecp2* KO neurons show significantly larger mEPSC amplitudes (Fig. 4C; *P*<0.001) and shorter inter-event intervals (IEIs) (Fig. 4B; *n*=11/4; *P*<0.01, K-S test), indicating that excitatory synapses onto CA1 pyramidal neurons are stronger by both pre- and postsynaptic mechanisms of quantal synaptic transmission. Consistent with its effects on the I-O relationship of VSD signals and fEPSPs, LM22A-4 treatment reduced the amplitude and increased the IEI of mEPSCs in *Mecp2* KO neurons (*n*=11/2) to WT levels, without any effects in WT neurons (*n*=11/4) (Fig. 4B,C; mEPSC amplitude: *P*<0.001, IEI: *P*<0.05 vs *Mecp2* KO control).

To confirm the effects of LM22A-4 on spontaneous neuronal activity, we imaged spontaneous Ca^2+^ transients (SCTs) in cultured hippocampal neurons [day in vitro (DIV) 7-13] filled with the Ca^2+^ indicator Fura-2 (Fig. 4D, Fig. S2A). To measure the effects of LM22A-4 on quantal excitatory synaptic transmission, Ca^2+^ imaging was performed in the presence of TTX (1 µM). Under these conditions, SCTs reflect the activation of voltage-gated calcium channels (VGCCs) during membrane depolarizations caused by temporally summing mEPSPs. Similar to whole-cell recordings of mEPSCs, the inter-event interval (IEI) of SCTs in *Mecp2* KO neurons was shorter compared with that in WT neurons (Fig. 4E,F; *P*<0.05, Student's *t*-test). Also consistent with its effects on mEPSCs, a single localized application of LM22A-4 to *Mecp2* KO neurons significantly increased the IEI of SCTs (*n*=12 cells/5 coverslips; *P*<0.05), but had no effect on WT neurons (Fig. 4E,F; *n*=9/3). This effect of LM22A-4 on SCT frequency is similar to that of locally applied BDNF to both WT and *Mecp2* KO neurons (Fig. S2B; WT BDNF, *n*=47/3; *Mecp2* KO BDNF, *n*=12/3; *P*<0.05, one-way ANOVA). Because these SCTs are due to all-or-none Ca^2+^ spikes driven by VGCCs, there were no differences between WT and *Mecp2* KO neurons or any effects of LM22A-4 or BDNF in SCT amplitude (360/380 nm ratio) (Fig. S2C). Further analysis showed that the half-width of SCTs was significantly shorter in *Mecp2* KO neurons, which was increased to WT levels after LM22A-4 or BDNF puffing (Fig. S2D; *P*<0.05). The effects on SCT duration are due to differences in decay time, but not rise time (Fig. S2E,F). Taken together, these results suggest that LM22A-4 reduces synaptic strength and tones down network hyperactivity, restoring the optimal range for synaptic plasticity.

## DISCUSSION

Several recent studies have indicated that the TrkB partial agonist LM22A-4 is a potentially useful therapeutic agent for several neurological diseases. LM22A-4 treatment was shown to promote motor recovery after hypoxic-ischemic stroke in mice (Han et al., 2012), improve motor impairment in the mouse model of Huntington's disease (Simmons et al., 2013), enhance the recovery of limb function after spinal cord injury in mice (Yu and Wang, 2015), reduce alcohol intake in the mouse model of BDNF polymorphism-related compulsive alcohol drinking (Warnault et al., 2016) and ameliorate respiratory abnormalities in a mouse model of RTT (Kron et al., 2014; Schmid et al., 2012). Our study provides additional support for the potential use of LM22A-4 in RTT by demonstrating that it reverts deficits in hippocampal-dependent object location memory and long-term synaptic plasticity in female *Mecp2* HET mice. LM22A-4 also reduces atypically stronger excitatory synaptic transmission and network hyperactivity in the hippocampus of female *Mecp2* HET mice, which is likely to restore the proper dynamic range of synaptic plasticity.

BDNF induces TrkB phosphorylation at mouse Tyr^515^ (equivalent to Tyr^490^ in mouse TrkA), Tyr^705/706^ and Tyr^816^ (equivalent to Tyr^817^ in human TrkB) in neurons (Minichiello, 2009). TrkB^Y515^ provides a docking site for Shc that leads to activation of PI3K/AKT and MAPK/ERK signaling pathways, TrkB^Y705/706^ is located in the activation loop that is responsible for TrkB autophosphorylation and TrkB^Y816^ creates a docking site for the PLCγ/PKC signaling cascade. As a partial agonist of TrkB receptors, LM22A-4 activates TrkB^Y515^, TrkB^Y705/706^ and TrkB^Y816^ both in control brains and in several mouse models of human neurological disorders (Han et al., 2012; Kajiya et al., 2014; Massa et al., 2010; Schmid et al., 2012; Simmons et al., 2013; Yu and Wang, 2015). Demonstrating central target engagement during our peripheral treatments with LM22A-4, TrkB^Y816^ is activated in the hippocampus of both WT and *Mecp2* HET mice, consistent with a robust increase in phosphorylation levels of its downstream target PLCγ. Interestingly, although TrkB phosphorylation can be induced by LM22A-4 in the WT hippocampus (Massa et al., 2010), it fails to do so in the WT brainstem (Schmid et al., 2012), suggesting that different brain regions have distinct sensitivity of TrkB phosphorylation sites, or that higher extracellular BDNF levels outcompete a partial agonist (see below).

The significant activation of PLCγ signaling in the hippocampus following LM22A-4 treatment may contribute to the restoration of spatial memory and LTP in female *Mecp2* HET mice, because PLCγ is necessary for LTP maintenance in area CA1 (Minichiello et al., 2002); however, LM22A-4 did not affect hippocampal function in WT mice. There are several possibilities why LM22A-4 could act differently in WT and *Mecp2* HET mice. First, BDNF levels are lower in *Mecp2*-deficient mice than in WT mice (Chang et al., 2006; Li et al., 2012; Schmid et al., 2012; Wang et al., 2006). In WT mice, the efficacy of LM22A-4 may be mitigated by the presence of the full TrkB agonist BDNF, while it can function in *Mecp2* HET mice. Second, TrkB receptors may have different sensitivity in response to LM22A-4 treatment. Increased neuronal activity in hippocampal cultures results in prolonged activation of TrkB and its downstream signaling (Guo et al., 2014). As in male *Mecp2* KO mice (Calfa et al., 2011a; Li et al., 2016), the hippocampus of female HET mice is hyperactive, which may alter the kinetics of TrkB signaling. Indeed, only acute slices from *Mecp2* KO mice showed increased TrkB phosphorylation after 30 min exposure to LM22A-4 (Fig. S1B). In addition, only hippocampal neurons from *Mecp2* KO mice responded to LM22A-4 by reducing the frequency of spontaneous Ca^2+^ transients. Third, altered network activity (hyper- or hypoactivity) may affect not only TrkB receptor sensitivity, but also local downstream signaling at synapses.

The short-term actions of BDNF on excitatory synaptic transmission vary depending on brain regions; for example, it: (1) increases evoked field EPSPs at CA3-CA1 synapses (Kang and Schuman, 1995; Ji et al., 2010); (2) decreases evoked EPSCs in sensory neurons within the nucleus of the tractus solitarius in the brainstem (Balkowiec et al., 2000); (3) decreases EPSCs in GABAergic neurons in the visual cortex (Jiang et al., 2004); (4) increases spontaneous quantal mEPSC frequency in hippocampal neurons (Lessmann et al., 1994; Amaral and Pozzo-Miller, 2012); and (5) has no effect at all on field EPSPs and evoked EPSCs at CA3-CA1 synapses (Patterson et al., 1996; Frerking et al., 1998; Gottschalk et al., 1998). Similarly, the long-term effects of BDNF vary; it: (1) increases EPSCs in autaptic cultures of hippocampal pyramidal neurons (Sherwood and Lo, 1999); (2) decreases AMPAR expression in medium spiny neurons of nucleus accumbens (Reimers et al., 2014); (3) increases spontaneous mEPSC frequency, synaptic vesicle docking at active zones, and spine density in hippocampal pyramidal neurons (Tyler and Pozzo-Miller, 2001); and (4) has no effect on evoked EPSCs in pyramidal neurons of rat visual cortical cultures (Rutherford et al., 1998). Such different actions of BDNF on excitatory synaptic transmission may be due to different levels of network activity in each brain region or experimental condition. For example, during prolonged neuronal activity induced by the GABA_A_ receptor antagonist bicuculine, the BDNF scavenger TrkB-Fc prevents scaling down of surface expression of AMPARs (Reimers et al., 2014). Furthermore, BDNF prevents the scaling up of mEPSC amplitude induced by prolonged activity blockade with TTX, while the BDNF scavenger TrkB-Fc mimics scaling-up of mEPSC amplitude during homeostatic synaptic plasticity (Rutherford et al., 1998). Similar to the higher levels of AMPAR during the homeostatic synaptic plasticity, *Mecp2*-deficient mice have higher surface levels of AMPARs at synapses, which saturates the dynamic range of synaptic plasticity (Li et al., 2016). The actions of LM22A-4 at hippocampal synapses of female *Mecp2* HET mice may reflect the restoration of activity-dependent AMPAR endocytosis, resulting in smaller evoked EPSPs, mEPSCs and spontaneous Ca^2+^ transients driven by mEPSPs. Alternatively, LM22A-4 may enhance TrkB-dependent maturation of GABAergic neurons and synapses, resulting in improved synaptic inhibition and hippocampal network stability.

In conclusion, we present evidence that chronic peripheral LM22A-4 treatment in female *Mecp2* HET mice improves motor function and hippocampal-dependent object location memory, and restores hippocampal long-term synaptic plasticity deficits. LM22A-4 exerts these effects by subduing excitatory synaptic transmission and network activity to levels amenable for the induction of synaptic plasticity and behavioral learning and memory. Our findings add to the growing body of literature supporting the high therapeutic potential of the TrkB ligand LM22A-4 for the treatment of RTT and other diseases associated with lower levels of BDNF.

## MATERIALS AND METHODS

### Animals

Breeding pairs of mice lacking exon 3 of *Mecp2* (B6.Cg-*Mecp2*^tm1.1Jae^, Jaenisch strain in a pure C57BL/6 background) (Chen et al., 2001) were purchased from the Mutant Mouse Regional Resource Center at the University of California, Davis. A colony was established at the University of Alabama at Birmingham by mating WT C57BL/6 male mice with heterozygous *Mecp2*^tm1.1Jae^ female mice (*Mecp2* HET), as recommended by the supplier. Genotyping was performed by PCR of DNA samples from tail clips. Hemizygous *Mecp2*^tm1.1Jae^ males (*Mecp2* KO), develop typically until 5-6 weeks of age, when they begin to exhibit RTT-like motor symptoms, such as hypoactivity, hind limb clasping and reflex impairments. Acute slices prepared from male symptomatic *Mecp2* KO mice were used for *in vitro* treatment. Female *Mecp2* HET mice, which develop RTT-like symptoms between 2-3 months of life (Samaco et al., 2013), were used for *in vivo* treatment because they represent the best model for preclinical studies (Katz et al., 2012). Animals were handled and housed according to the Committee on Laboratory Animal Resources of the National Institutes of Health; all experimental protocols were reviewed annually and approved by the Institutional Animals Care and Use Committee of the University of Alabama at Birmingham.

### *In vivo* LM22A-4 treatment

LM22A-4 was prepared fresh daily. Female *Mecp2* HET mice and their age-matched WT littermates (∼4 months old) received intraperitoneal (i.p.) injections of either sterile LM22A-4 (50 mg/kg) or vehicle (0.9% NaCl) twice daily for 1 or 2 months, following an established dosing regime (Schmid et al., 2012). Mice were randomly assigned to each treatment.

### General phenotype

General phenotype was evaluated according to an established protocol (Guy et al., 2007). We visually scored RTT-like general symptoms consisting of ill-groomed condition, reduced motility, imbalanced gait, hind limb clasping, irregular breathing and tremor. Each category was scored every week during the treatment with LM22A-4 or vehicle according to their symptom severity (0: absent; 1: moderate; 2: severe), and the aggregated value was then calculated to reflect general phenotypes during disease progression. We scored the following features: (A) General condition; 0: clean and sleek hair, limpid eyes; 1: ungroomed hair, opaque eyes; 2: severe piloerection, narrowing eye. (B) Motility; 0: free and steady movement; 1: less movement, frequent freezing; 2: almost no voluntary movement. (C) Gait; 0: normal stance; 1: wider spread during the movement of the hind legs; 2: severe walking abnormality, low pelvic elevation. (D) Hind limb clasping; 0: legs spreading outwards; 1: one leg frequently drawing close to the body; 2: two legs always tightening close to the body. (E) Breathing; 0: normal breathing; 1: occasional breathing stops and gasping; 2: frequent breathing stops and gasping. (F) Tremor; 0: no tremor; 1: occasional mild tremor; 2: continuous tremor.

### Open field test

WT and *Mecp2* HET mice that received either LM22A-4 or vehicle for 2 months were placed in the center of a 60×60 cm open arena. Following 10 min habituation, mice were imaged for 10 min with an IR-sensitive Gigabit Ethernet video camera (ace acA780-75gm, Basler). Recorded activity was analyzed for the distance that these mice traveled. The open field arena was cleaned with 70% ethanol between each trial.

### Dowel crossings

Control or LM22A-4-treated mice were placed on the center of a dowel suspended between two platforms. The time to successfully cross the dowel without falling off was recorded for each mouse. If the mouse failed, the mouse was placed back to the center of the dowel. The test lasted 2 min beginning when the mouse was placed on the dowel.

### Object location test

The object location test for mice was adapted from an established protocol (Murai et al., 2007). Mice were habituated for 3 consecutive days before testing, by allowing them to freely explore the empty test field (30×40 cm) for 5 min. During the acquisition phase, one mouse was allowed 5 min to freely explore 2 identical objects placed equidistant from each other and from the walls. The mouse was then returned to its home cage for a 2 h consolidation phase. During the test trial, the mouse was returned to the open field where one object had been moved to a new location, and allowed to freely explore the objects for 5 min. The new location of the displaced object was counterbalanced for each mouse to prevent the use of spatial cues outside the test field. The acquisition and test phases were imaged with an IR-sensitive Gigabit Ethernet video camera and movies saved directly to a hard disk for offline analyses (ImageJ). The time spent exploring each object in the test phase was scored to calculate a location preference index as *T*_d_×100/*T*_nd_+*T*_d_, where *T*_d_ is the time spent exploring the displaced object, and *T*_nd_ the time spent exploring the non-displaced object. Exploration was defined as pointing the nose towards the object at a distance of <1 cm and/or touching with the nose; circling or climbing the object was not considered exploration.

### Western immunoblotting

For short-term drug treatment in acute brain slices, mice were anesthetized with isoflurane, and the brain was rapidly removed and placed in ice-cold cutting artificial cerebrospinal fluid (aCSF) (87 mM NaCl, 2.5 mM KCl, 0.5 mM CaCl_2_, 7 mM MgCl_2_, 1.25 mM NaH_2_PO_4_, 25 mM NaHCO_3_, 25 mM glucose and 75 mM sucrose, bubbled with 95% O_2_/5% CO_2_). The brain was cut transversely at 300 μm using a vibrating blade microtome (VT1200S, Leica) and slices were transferred to standard aCSF (125 mM NaCl, 2.5 mM KCl, 2 mM CaCl_2_, 1 mM MgCl_2_, 1.25 mM NaH_2_PO_4_, 25 mM NaHCO_3_ and 25 mM glucose, bubbled with 95% O_2_/5% CO_2_) at 32°C for 30 min, and then allowed to recover at room temperature (24°C) for 1 h prior to drug exposure. Slices were randomly assigned to treatment groups: control, LM22A-4 (500 nM), recombinant human BDNF (250 ng/ml, Promega, G1491), K252a (200 nM, Calbiochem, 420298), LM22A-4+K252a, and BDNF+K252a. Two to four slices per mouse were perfused under each treatment condition at 4-6 ml/min for 30 min. Following treatment, the hippocampus was quickly dissected from each slice and rapidly frozen on dry ice. For sample preparation from female WT and *Mecp2* Het mice receiving *in vivo* treatment, hippocampi were dissected after the last injection and rapidly frozen on dry ice.

Hippocampal samples were homogenized with a sonicator on ice in Nonidet P-40 buffer (20 mM Tris-HCl at pH 8.0, 137 mM NaCl, 10% glycerol, 1% Nonidet P-40, 2 mM EDTA) containing protease inhibitor and phosphatase inhibitor (Sigma). The homogenates were agitated for 2 h and then centrifuged at 12,000 ***g*** for 20 min at 4°C. The supernatants were aspirated and protein concentrations determined by the Lowry method. Equal amounts of protein sample were denatured in loading buffer (125 mM Tris-HCl at pH 6.8, 20% glycerol, 6% SDS, and 5% 2-mercaptoethanol), boiled for 3 min, and subject to SDS-PAGE. Proteins were then transferred to PVDF membranes and blocked with 5% nonfat milk in TBST (20 mM Tris-HCl at pH 7.6, 150 mM NaCl and 0.1% Tween-20) for 1 h. Membranes were incubated with primary antibodies against phospho-TrkB^Y816^ (1:500, Abcam, ab75173), total-TrkB (1:200, Santa Cruz, SC20542), phospho-ERK (1:1000, Cell Signaling, 9101S), total ERK (1:2000, Cell Signaling, 4695S), phospho-AKT (1:1000, Cell Signaling, 9271S), total AKT (1:2000, Cell Signaling, 9272S), phospho-PLCγ (1:250, Cell Signaling, 8713S), and total PLCγ (1:1000, Cell Signaling, 2822S), and then with corresponding HRP-conjugated secondary antibodies (Santa Cruz). The protein bands were detected using the Pierce ECL Substrate (Thermo Fisher Scientific) and signals were captured on autoradiography film. The membranes were re-probed for the loading control with β-actin (Thermo Fisher Scientific) and detected using an Odyssey infrared imaging system after incubation with fluorescent secondary antibodies (LI-COR Bioscience). Protein levels of bands were quantified using computer-assisted densitometry; only non-saturated bands were analyzed. Ratios of all bands to the loading controls were obtained, values were normalized to control, and then the ratios of phospho-TrkB to full-length TrkB, phospho-ERK to total ERK, phospho-AKT to total AKT, and phospho-PLCγ to total PLCγ were calculated as measures of activation of TrkB receptors and their intracellular signaling cascade.

### Organotypic slice cultures

Slice cultures were prepared from male *Mecp2* KO mice and WT littermates at postnatal day 5-7 (P5-7), as described (Chapleau et al., 2009). Hippocampal slices 500 µm thick were plated on tissue culture plate inserts (Millicell-CM) in 6-well plates with serum-containing culture medium consisting of Neurobasal-A without Phenol Red (Invitrogen), 20% heat-inactivated equine serum (Invitrogen), B27 supplement (Invitrogen), 0.5 mM L-glutamine (Invitrogen) and placed in an incubator at 36°C, 5% CO_2_, 90% relative humidity. Serum was titrated out over 3 days *in vitro* and treatments were made in serum-free culture medium (Chapleau et al., 2008). At DIV 10-12, medium was aspirated from culture wells and replaced with serum-free medium containing LM22A-4 (500 nM), followed by gentle application of 50 μl of drug-containing medium on top of each hippocampal slice. After 2 days of LM22A-4 treatment, slices were transferred to an immersion chamber for whole-cell patch clamping.

### High-speed VSD imaging and electrophysiology

Acute hippocampal slices from female WT and *Mecp2* HET mice that received treatment for 2 months were prepared as described above. After recovery, individual slices were stained with the voltage-sensitive fluorescent dye RH414 (30 µM in aCSF, Anaspec) for 1 h at room temperature, and transferred to an immersion chamber continuously perfused (2 ml/min) with aCSF at 32°C and saturated with 95%O_2_/5%CO_2_. VSD signals and extracellular field EPSPs were evoked in CA1 stratum radium by stimulation of Schaffer collaterals. RH414 was excited at 530±50 nm with a phosphor-pumped LED (Heliophor, 89North), and its filtered fluorescence (535±50 nm band-pass, 580 nm beam-splitter, 594 nm long-pass, Semrock) imaged in an inverted microscope (IX71, Olympus) through a 10×0.5NA objective (Fluar, Zeiss) and acquired with a scientific CMOS camera running at 2500 frames per second in full 128×128 pixel resolution (NeuroCMOS-SM128, RedShirt Imaging). Field EPSPs were acquired with Axopatch-2A amplifier (Molecular Devices) in current-clamp mode, filtered at 2 kHz, and digitized at 10 kHz with ITC-18 A/D-D/A interface (Instrutech) controlled by custom-written software on a G5 PowerMac computer (TI-WorkBench, provided by Dr Takafumi Inoue, Waseda University, Tokyo). The I-O relationship of VSD signals and field EPSPs was obtained by delivering three different stimulus intensities with 30 µA increments. LTP of VSD signals and field EPSPs was induced by TBS of afferent fibers, which consisted of 4 trains of 10 bursts of 5 pulses at 100 Hz, with 200 ms between bursts (5 Hz), and 5 s between trains. The peak amplitude of VSD signals was measured in a 3×3 pixel region. The cumulative percentage of VSD amplitude and VSD spatial spread was obtained by measuring the area showing ΔF/F levels 2-times the baseline noise.

mEPSCs were recorded in the whole-cell configuration from CA1 pyramidal neurons within hippocampal slice cultures at a holding membrane voltage of −60 mV in the presence of the sodium channel blocker TTX (1 µM) and the GABA_A_R antagonist picrotoxin (50 µM). mEPSCs were analyzed using MiniAnalysis (Synaptosoft), with a detection threshold of 6 pA. The cumulative probability distribution of mEPSC amplitudes and inter-event intervals were calculated.

### Primary cultures of hippocampal neurons and intracellular Ca^2+^ imaging

Primary neuronal cultures were obtained from anesthetized postnatal day 0 or 1 (P0-1) male *Mecp2* KO mice and WT littermates. Both hippocampi were dissociated in papain (20 U/ml) plus DNase I (Worthington, Lakewood, NJ, USA) for 20-30 min at 37°C, as described (Amaral and Pozzo-Miller, 2007). The tissue was then triturated to obtain a single-cell suspension, and the cells were plated at a density of 50,000 cells/cm^2^ on 12 mm poly-L-lysine/laminin-coated glass coverslips, and immersed in Neurobasal medium supplemented with 2% B27 and 0.5 mM glutamine (Life Technologies, Carlsbad, CA). Neurons were grown in 37°C, 5% CO_2_, 90% relative humidity incubators (Thermo-Forma), with half of the fresh culture medium changed every 3-4 days.

Fura-2 AM (Invitrogen) was dissolved in 20% pluronic acid/DMSO, and Fura-2 AM stock solution (5 mM) was then diluted in aCSF to obtain the working solution (5 µM). Neurons (DIV 7-13) on the coverslips were incubated with Fura-2 AM at room temperature for 30 min. The coverslips were then transferred to a recording chamber and continuously perfused with aCSF (1 ml/min). Neurons were imaged in a fluorescence microscope with a 40×0.75NA water immersion objective. A 16 min movie with 4 s frame intervals was taken by exciting Fura-2 AM with 360 nm and 380 nm light from a monochromator (Polychrome-II, TILL Photonics), and its emission at >510 nm was detected with a cooled CCD camera (CoolSNAP, Photometrics). Ca^2+^ signals were imaged in the presence of TTX (1 μM) to block voltage-gated Na^+^ channels. LM22A-4 (500 nM) or BDNF (250 ng/ml) were pressure-applied (2 min, 2 pounds per square inch) to WT or *Mecp2* KO neurons during imaging. Regions of interest (ROIs) were drawn on individual neuronal cell bodies for Ca^2+^ signals and a cell-free region for background subtraction. Spontaneous Ca^2+^ transients (SCTs) in the 360/380 nm ratio were automatically detected and analyzed using custom-written codes in MATLAB (MathWorks). The inter-event interval, amplitude, half-width, rise time and decay time of SCTs were calculated.

### Statistical analyses

All statistical analyses were performed blinded to the genotype and treatment groups using Prism (GraphPad), MATLAB, and G*Power. Data were compared using Student's *t*-test for two groups and one-way ANOVA with Bonferroni's *post hoc* test for more than two groups. The time courses of mouse weight and general phenotype score were analyzed using two-way repeated measures ANOVA. The Kolmogorov–Smirnov (K-S) test was used for comparisons of cumulative percentage distributions. *P*<0.05 was considered significant.

## References

[DMM029959C1] Abuhatzira L., Makedonski K., Kaufman Y., Razin A. and Shemer R. (2007). MeCP2 deficiency in the brain decreases BDNF levels by REST/CoREST-mediated repression and increases TRKB production. *Epigenetics* 2, 214-222. 10.4161/epi.2.4.5212 18075316

[DMM029959C2] Amaral M. D. and Pozzo-Miller L. (2007). TRPC3 channels are necessary for brain-derived neurotrophic factor to activate a nonselective cationic current and to induce dendritic spine formation. *J. Neurosci.* 27, 5179-5189. 10.1523/jneurosci.5499-06.2007 17494704 PMC2806846

[DMM029959C3] Amaral M. D. and Pozzo-Miller L. (2012). Intracellular Ca^2+^ stores and Ca^2+^ influx are both required for BDNF to rapidly increase quantal vesicular transmitter release. *Neural Plast.* 2012, 203536 10.1155/2012/203536 22811938 PMC3397209

[DMM029959C4] Amir R. E., Van den Veyver I. B., Wan M., Tran C. Q., Francke U. and Zoghbi H. Y. (1999). Rett syndrome is caused by mutations in X-linked MECP2, encoding methyl-CpG-binding protein 2. *Nat. Genet.* 23, 185-188. 10.1038/13810 10508514

[DMM029959C5] Balkowiec A., Kunze D. L. and Katz D. M. (2000). Brain-derived neurotrophic factor acutely inhibits AMPA-mediated currents in developing sensory relay neurons. *J. Neurosci.* 20, 1904-1911. 10684891 10.1523/JNEUROSCI.20-05-01904.2000 PMC6772909

[DMM029959C6] Barker G. R. I. and Warburton E. C. (2011). When is the hippocampus involved in recognition memory? *J. Neurosci.* 31, 10721-10731. 10.1523/JNEUROSCI.6413-10.2011 21775615 PMC6622630

[DMM029959C7] Calfa G., Hablitz J. J. and Pozzo-Miller L. (2011a). Network hyperexcitability in hippocampal slices from Mecp2 mutant mice revealed by voltage-sensitive dye imaging. *J. Neurophysiol.* 105, 1768-1784. 10.1152/jn.00800.2010 21307327 PMC3075283

[DMM029959C8] Calfa G., Percy A. K. and Pozzo-Miller L. (2011b). Experimental models of Rett syndrome based on *Mecp2* dysfunction. *Exp. Biol. Med.* 236, 3-19. 10.1258/ebm.2010.010261 PMC3059199 21239731

[DMM029959C9] Chahrour M., Jung S. Y., Shaw C., Zhou X., Wong S. T. C., Qin J. and Zoghbi H. Y. (2008). MeCP2, a key contributor to neurological disease, activates and represses transcription. *Science* 320, 1224-1229. 10.1126/science.1153252 18511691 PMC2443785

[DMM029959C10] Chang P. Y. and Jackson M. B. (2006). Heterogeneous spatial patterns of long-term potentiation in rat hippocampal slices. *J. Physiol.* 576, 427-443. 10.1113/jphysiol.2006.112128 16873414 PMC1890346

[DMM029959C11] Chang Q., Khare G., Dani V., Nelson S. and Jaenisch R. (2006). The disease progression of *Mecp2* mutant mice is affected by the level of BDNF expression. *Neuron* 49, 341-348. 10.1016/j.neuron.2005.12.027 16446138

[DMM029959C12] Chapleau C. A., Carlo M. E., Larimore J. L. and Pozzo-Miller L. (2008). The actions of BDNF on dendritic spine density and morphology in organotypic slice cultures depend on the presence of serum in culture media. *J. Neurosci. Methods* 169, 182-190. 10.1016/j.jneumeth.2007.12.006 18242714 PMC2348185

[DMM029959C13] Chapleau C. A., Calfa G. D., Lane M. C., Albertson A. J., Larimore J. L., Kudo S., Armstrong D. L., Percy A. K. and Pozzo-Miller L. (2009). Dendritic spine pathologies in hippocampal pyramidal neurons from Rett syndrome brain and after expression of Rett-associated *MECP2* mutations. *Neurobiol. Dis.* 35, 219-233. 10.1016/j.nbd.2009.05.001 19442733 PMC2722110

[DMM029959C14] Chen R. Z., Akbarian S., Tudor M. and Jaenisch R. (2001). Deficiency of methyl-CpG binding protein-2 in CNS neurons results in a Rett-like phenotype in mice. *Nat. Genet.* 27, 327-331. 10.1038/85906 11242118

[DMM029959C15] Chen W. G., Chang Q., Lin Y., Meissner A., West A. E., Griffith E. C., Jaenisch R. and Greenberg M. E. (2003). Derepression of BDNF transcription involves calcium-dependent phosphorylation of MeCP2. *Science* 302, 885-889. 10.1126/science.1086446 14593183

[DMM029959C16] Deng V., Matagne V., Banine F., Frerking M., Ohliger P., Budden S., Pevsner J., Dissen G. A., Sherman L. S. and Ojeda S. R. (2007). FXYD1 is an MeCP2 target gene overexpressed in the brains of Rett syndrome patients and *Mecp2*-null mice. *Hum. Mol. Genet.* 16, 640-650. 10.1093/hmg/ddm007 17309881

[DMM029959C17] Frerking M., Malenka R. C. and Nicoll R. A. (1998). Brain-derived neurotrophic factor (BDNF) modulates inhibitory, but not excitatory, transmission in the CA1 region of the hippocampus. *J. Neurophysiol.* 80, 3383-3386. 9862938 10.1152/jn.1998.80.6.3383

[DMM029959C18] Gottschalk W., Pozzo-Miller L. D., Figurov A. and Lu B. (1998). Presynaptic modulation of synaptic transmission and plasticity by brain-derived neurotrophic factor in the developing hippocampus. *J. Neurosci.* 18, 6830-6839. 9712654 10.1523/JNEUROSCI.18-17-06830.1998 PMC6792976

[DMM029959C19] Guo W., Ji Y., Wang S., Sun Y. and Lu B. (2014). Neuronal activity alters BDNF–TrkB signaling kinetics and downstream functions. *J. Cell Sci.* 127, 2249-2260. 10.1242/jcs.139964 24634513

[DMM029959C20] Guy J., Gan J., Selfridge J., Cobb S. and Bird A. (2007). Reversal of neurological defects in a mouse model of Rett syndrome. *Science* 315, 1143-1147. 10.1126/science.1138389 17289941 PMC7610836

[DMM029959C21] Han J., Pollak J., Yang T., Siddiqui M. R., Doyle K. P., Taravosh-Lahn K., Cekanaviciute E., Han A., Goodman J. Z., Jones B. et al. (2012). Delayed administration of a small molecule tropomyosin-related kinase B ligand promotes recovery after hypoxic-ischemic stroke. *Stroke* 43, 1918-1924. 10.1161/STROKEAHA.111.641878 22535263 PMC3383889

[DMM029959C22] Ji Y., Lu Y., Yang F., Shen W., Tang T. T.-T., Feng L., Duan S. and Lu B. (2010). Acute and gradual increases in BDNF concentration elicit distinct signaling and functions in neurons. *Nat. Neurosci.* 13, 302-309. 10.1038/nn.2505 20173744 PMC4780419

[DMM029959C23] Jiang B., Kitamura A., Yasuda H., Sohya K., Maruyama A., Yanagawa Y., Obata K. and Tsumoto T. (2004). Brain-derived neurotrophic factor acutely depresses excitatory synaptic transmission to GABAergic neurons in visual cortical slices. *Eur. J. Neurosci.* 20, 709-718. 10.1111/j.1460-9568.2004.03523.x 15255981

[DMM029959C24] Kajiya M., Takeshita K., Kittaka M., Matsuda S., Ouhara K., Takeda K., Takata T., Kitagawa M., Fujita T., Shiba H. et al. (2014). BDNF mimetic compound LM22A-4 regulates cementoblast differentiation via the TrkB-ERK/Akt signaling cascade. *Int. Immunopharmacol.* 19, 245-252. 10.1016/j.intimp.2014.01.028 24530412

[DMM029959C25] Kang H. and Schuman E. M. (1995). Long-lasting neurotrophin-induced enhancement of synaptic transmission in the adult hippocampus. *Science* 267, 1658-1662. 10.1126/science.7886457 7886457

[DMM029959C26] Katz D. M. (2014). Brain-derived neurotrophic factor and Rett syndrome. *Handb. Exp. Pharmacol.* 220, 481-495. 10.1007/978-3-642-45106-5_18 24668484

[DMM029959C27] Katz D. M., Berger-Sweeney J. E., Eubanks J. H., Justice M. J., Neul J. L., Pozzo-Miller L., Blue M. E., Christian D., Crawley J. N., Giustetto M. et al. (2012). Preclinical research in Rett syndrome: setting the foundation for translational success. *Dis. Model. Mech.* 5, 733-745. 10.1242/dmm.011007 23115203 PMC3484856

[DMM029959C28] Kron M., Lang M., Adams I. T., Sceniak M., Longo F. and Katz D. M. (2014). A BDNF loop-domain mimetic acutely reverses spontaneous apneas and respiratory abnormalities during behavioral arousal in a mouse model of Rett syndrome. *Dis. Model. Mech.* 7, 1047-1055. 10.1242/dmm.016030 25147297 PMC4142725

[DMM029959C29] Lauterborn J. C., Lynch G., Vanderklish P., Arai A. and Gall C. M. (2000). Positive modulation of AMPA receptors increases neurotrophin expression by hippocampal and cortical neurons. *J. Neurosci.* 20, 8-21. 10627576 10.1523/JNEUROSCI.20-01-00008.2000 PMC6774091

[DMM029959C30] Lessmann V., Gottmann K. and Heumann R. (1994). BDNF, and NT-4/5 enhance glutamatergic synaptic transmission in cultured hippocampal neurones. *Neuroreport* 6, 21-25. 10.1097/00001756-199412300-00007 7703418

[DMM029959C31] Li W. and Pozzo-Miller L. (2012). Beyond widespread *Mecp2* deletions to model Rett syndrome: conditional spatio-temporal knockout, single-point mutations and transgenic rescue mice. *Autism Open Access* 2012 (Suppl. 1), 5 10.4172/2165-7890.S1-005 23946910 PMC3740402

[DMM029959C32] Li W., Calfa G., Larimore J. and Pozzo-Miller L. (2012). Activity-dependent BDNF release and TRPC signaling is impaired in hippocampal neurons of *Mecp2* mutant mice. *Proc. Natl. Acad. Sci. USA* 109, 17087-17092. 10.1073/pnas.1205271109 23027959 PMC3479462

[DMM029959C33] Li W., Xu X. and Pozzo-Miller L. (2016). Excitatory synapses are stronger in the hippocampus of Rett syndrome mice due to altered synaptic trafficking of AMPA-type glutamate receptors. *Proc. Natl. Acad. Sci. USA* 113, E1575-E1584. 10.1073/pnas.1517244113 26929363 PMC4801299

[DMM029959C34] Martinowich K., Hattori D., Wu H., Fouse S., He F., Hu Y., Fan G. and Sun Y. E. (2003). DNA methylation-related chromatin remodeling in activity-dependent BDNF gene regulation. *Science* 302, 890-893. 10.1126/science.1090842 14593184

[DMM029959C35] Massa S. M., Yang T., Xie Y., Shi J., Bilgen M., Joyce J. N., Nehama D., Rajadas J. and Longo F. M. (2010). Small molecule BDNF mimetics activate TrkB signaling and prevent neuronal degeneration in rodents. *J. Clin. Invest.* 120, 1774-1785. 10.1172/JCI41356 20407211 PMC2860903

[DMM029959C36] Minichiello L. (2009). TrkB signalling pathways in LTP and learning. *Nat. Rev. Neurosci.* 10, 850-860. 10.1038/nrn2738 19927149

[DMM029959C37] Minichiello L., Calella A. M., Medina D. L., Bonhoeffer T., Klein R. and Korte M. (2002). Mechanism of TrkB-mediated hippocampal long-term potentiation. *Neuron* 36, 121-137. 10.1016/S0896-6273(02)00942-X 12367511

[DMM029959C38] Murai T., Okuda S., Tanaka T. and Ohta H. (2007). Characteristics of object location memory in mice: Behavioral and pharmacological studies. *Physiol. Behav.* 90, 116-124. 10.1016/j.physbeh.2006.09.013 17049363

[DMM029959C39] Neul J. L., Kaufmann W. E., Glaze D. G., Christodoulou J., Clarke A. J., Bahi-Buisson N., Leonard H., Bailey M. E. S., Schanen N. C., Zappella M. et al.; RettSearch Consortium. (2010). Rett syndrome: revised diagnostic criteria and nomenclature. *Ann. Neurol.* 68, 944-950. 10.1002/ana.22124 21154482 PMC3058521

[DMM029959C40] Ogier M., Wang H., Hong E., Wang Q., Greenberg M. E. and Katz D. M. (2007). Brain-derived neurotrophic factor expression and respiratory function improve after ampakine treatment in a mouse model of Rett syndrome. *J. Neurosci.* 27, 10912-10917. 10.1523/JNEUROSCI.1869-07.2007 17913925 PMC6672830

[DMM029959C41] Park H. and Poo M.-M. (2013). Neurotrophin regulation of neural circuit development and function. *Nat. Rev. Neurosci.* 14, 7-23. 10.1038/nrn3379 23254191

[DMM029959C42] Patterson S. L., Abel T., Deuel T. A. S., Martin K. C., Rose J. C. and Kandel E. R. (1996). Recombinant BDNF rescues deficits in basal synaptic transmission and hippocampal LTP in BDNF knockout mice. *Neuron* 16, 1137-1145. 10.1016/S0896-6273(00)80140-3 8663990

[DMM029959C43] Percy A. K. (2011). Rett syndrome: exploring the autism link. *Arch. Neurol.* 68, 985-989. 10.1001/archneurol.2011.149 21825235 PMC3674963

[DMM029959C44] Reimers J. M., Loweth J. A. and Wolf M. E. (2014). BDNF contributes to both rapid and homeostatic alterations in AMPA receptor surface expression in nucleus accumbens medium spiny neurons. *Eur. J. Neurosci.* 39, 1159-1169. 10.1111/ejn.12422 24712995 PMC4410784

[DMM029959C45] Rutherford L. C., Nelson S. B. and Turrigiano G. G. (1998). BDNF has opposite effects on the quantal amplitude of pyramidal neuron and interneuron excitatory synapses. *Neuron* 21, 521-530. 10.1016/S0896-6273(00)80563-2 9768839

[DMM029959C46] Samaco R. C., McGraw C. M., Ward C. S., Sun Y., Neul J. L. and Zoghbi H. Y. (2013). Female *Mecp2*^+/−^ mice display robust behavioral deficits on two different genetic backgrounds providing a framework for pre-clinical studies. *Hum. Mol. Genet.* 22, 96-109. 10.1093/hmg/dds406 23026749 PMC3522402

[DMM029959C47] Schmid D. A., Yang T., Ogier M., Adams I., Mirakhur Y., Wang Q., Massa S. M., Longo F. M. and Katz D. M. (2012). A TrkB small molecule partial agonist rescues TrkB phosphorylation deficits and improves respiratory function in a mouse model of Rett syndrome. *J. Neurosci.* 32, 1803-1810. 10.1523/JNEUROSCI.0865-11.2012 22302819 PMC3710112

[DMM029959C48] Sherwood N. T. and Lo D. C. (1999). Long-term enhancement of central synaptic transmission by chronic brain-derived neurotrophic factor treatment. *J. Neurosci.* 19, 7025-7036. 10436057 10.1523/JNEUROSCI.19-16-07025.1999 PMC6782869

[DMM029959C49] Simmons D. A., Belichenko N. P., Yang T., Condon C., Monbureau M., Shamloo M., Jing D., Massa S. M. and Longo F. M. (2013). A small molecule TrkB ligand reduces motor impairment and neuropathology in R6/2 and BACHD mouse models of Huntington's disease. *J. Neurosci.* 33, 18712-18727. 10.1523/JNEUROSCI.1310-13.2013 24285878 PMC3841443

[DMM029959C50] Tyler W. J. and Pozzo-Miller L. D. (2001). BDNF enhances quantal neurotransmitter release and increases the number of docked vesicles at the active zones of hippocampal excitatory synapses. *J. Neurosci.* 21, 4249-4258. 11404410 10.1523/JNEUROSCI.21-12-04249.2001 PMC2806848

[DMM029959C51] Wang H., Chan S.-A., Ogier M., Hellard D., Wang Q., Smith C. and Katz D. M. (2006). Dysregulation of brain-derived neurotrophic factor expression and neurosecretory function in *Mecp2* null mice. *J. Neurosci.* 26, 10911-10915. 10.1523/JNEUROSCI.1810-06.2006 17050729 PMC6674736

[DMM029959C52] Warnault V., Darcq E., Morisot N., Phamluong K., Wilbrecht L., Massa S. M., Longo F. M. and Ron D. (2016). The BDNF valine 68 to methionine polymorphism increases compulsive alcohol drinking in mice that is reversed by tropomyosin receptor kinase B activation. *Biol. Psychiatry* 79, 463-473. 10.1016/j.biopsych.2015.06.007 26204799 PMC4676961

[DMM029959C53] Yu G. and Wang W. (2015). Protective effects of LM22A-4 on injured spinal cord nerves. *Int. J. Clin. Exp. Pathol.* 8, 6526-6532. 26261531 PMC4525865

